# Tuberculosis recurrences and predictive factors in a vulnerable population in Catalonia

**DOI:** 10.1371/journal.pone.0227291

**Published:** 2020-01-15

**Authors:** Sílvia Brugueras, Vinicio-Israel Molina, Xavier Casas, Yoel-Domingo González, Nuria Forcada, Dora Romero, Anna Rodés, Maria-Neus Altet, José Maldonado, Mario Martin-Sánchez, Joan A. Caylà, Àngels Orcau, Cristina Rius, Joan-Pau Millet

**Affiliations:** 1 Epidemiology Service, Agència de Salut Pública de Barcelona (ASPB), Barcelona, Spain; 2 Consorcio de Investigación Biomédica en Red de Epidemiología y Salud Pública (CIBERESP), Madrid, Spain; 3 Departamento de Pediatría, Obstetricia y Ginecología y Medicina Preventiva, Facultad de Medicina, Universidad Autónoma de Barcelona, Barcelona, Spain; 4 Serveis Clínics, Barcelona, Spain; 5 Agència de Salut Pública de Catalunya, Barcelona, Spain; 6 Preventive Medicine and Public Health Training Unit Parc de Salut Mar–Pompeu Fabra University—Public Health Agency of Barcelona (PSMar-UPF-ASPB), Barcelona, Spain; 7 Foundation of the Tuberculosis Research Unit of Barcelona (fuiTB), Barcelona, Spain; Institute of Tropical Medicine Antwerp, BELGIUM

## Abstract

**Background:**

Patients with a history of tuberculosis (TB) have a high probability of recurrence because long-term cure is not always maintained in successfully treated patients. The aim of this study was to identify the probability of TB recurrence and its predictive factors in a cohort of socially vulnerable patients who completed treatment in the TB referral center in Catalonia, which acts as the center for patients with social and health problems.

**Methods:**

This retrospective open cohort study included all patients diagnosed with TB who were admitted and successfully treated in Serveis Clínics between 2000 and 2016 and who remained disease-free for a minimum of 1 year after treatment completion. We calculated the incidence density of TB recurrences per person-years of follow-up. We also estimated the cumulative incidence of TB recurrence at 1, 2, 5, and 10 years of follow-up. Bivariate analysis was conducted using Kaplan-Meier curves. Multivariate analysis was conducted using Cox regression. Hazard ratios (HR) were calculated with their 95% confidence intervals (95%CI).

**Results:**

There were 839 patients and 24 recurrences (2.9%), representing 0.49 per 100 person-years. The probability of a recurrence was 0.63% at 1 year of follow-up, 1.35% at 2 years, and 3.69% at 5 years. The multivariate analysis showed that the predictive factors of recurrence were age older than 34 years (aHR = 3.90; CI = 1.06–14.34 at age 35–45 years and aHR = 3.88; CI = 1.02–14.80 at age >45 years) and resistance to at least one anti-TB drug (aHR = 2.91; CI = 1.11–7.65).

**Conclusions:**

Attention should be paid to socially vulnerable persons older than 34 years with a previous episode of resistant TB. Surveillance resources should be directed toward adequately treated patients who nevertheless have a high risk of recurrence.

## Introduction

In 2017, there were an estimated 10 million people with tuberculosis (TB) worldwide. Although considerable progress has been made in the last few years, the main obstacles to controlling the infection are human immunodeficiency virus (HIV), the increase in antibiotic resistance, and the problem of TB in large cities associated with socially vulnerable populations at risk of social exclusion, such as drug users, alcoholics, and economic immigrants [1–4]. Interest in TB recurrences has been growing because, despite efforts to control the disease globally, long-term cure is not maintained in all successfully treated patients [5,6]. Compared with patients without a prior TB episode, those with a history of TB are at higher risk of contracting the disease again [7].

Tuberculosis recurrence can be defined as a new episode occurring in adequately treated patients who completed therapy [8]. Both the Centers for Disease Control and the Instituto de Salud Carlos III (ISCIII) accept that all TB episodes occurring 1 year or later after treatment completion in patients who were therefore disease-free should be considered as new TB cases and consequently as recurrences [9,10].

In areas with a low incidence of TB, most recurrences are attributed to reactivation or incomplete cure of the first TB episode, instead of a new infection due to repeat exposure. In contrast, the latter is more common in countries with a high burden of the disease, where transmission is highly frequent [5,11–13].

While the factors associated with poor treatment adherence are well known, there are few reports of recurrence rates and their associated factors in vulnerable populations in countries with a medium or low incidence [5,6,14,15]. Such knowledge would help to identify the populations that are most susceptible and which have the highest risk of recurrent TB. This would allow the design of strategies and help to focus efforts and interventions on post-treatment follow-up and application of public health measures to minimize the risk of a new episode.

In Catalonia, directly observed treatment (DOT) is not indicated in all TB patients. DOT is indicated in patients with risk factors for treatment non-adherence (alcohol or substance abuse, homelessness or immigration from countries with a high TB burden) or those with antibiotic resistance [16]. Serveis Clínics is a center providing community-based DOT and also has a TB clinic that is the referral center for the whole of Catalonia and serves as the center for patients with risk factors, poor adherence, multidrug resistant TB (MDR-TB) or extensively drug-resistant TB (XDR).

The aim of this study was to determine the probability of TB recurrence and its predictive factors in a cohort of especially vulnerable patients who completed treatment in Servies Clínics.

## Methods

### Study design and setting

This retrospective, open cohort study included all patients with TB admitted to Serveis Clínics and who started treatment between 2000 and 2016.

### Study population

The study population consisted of patients with a diagnosis of TB, who were successfully treated and disease-free a minimum of 1 year after completing treatment and who started treatment after 1st January, 2000. Patient inclusion ended with patients who were successfully treated up to 30th October, 2016. The patients were retrospectively followed-up until 30th October, 2017. Length of follow-up was calculated bearing in mind the time interval between treatment end and 1 year until recurrence, death, change of address, or end of the study. A flow diagram of included patients and recurrences is shown in S1 Fig.

### Definitions

TB recurrence was defined as the presence of a new episode of the disease after completion of adequate treatment in persons considered to have been successfully treated and disease-free for a minimum of 1 year after the end of treatment [9,10].

A cured patient was a patient whose sputum smear or culture was positive at the beginning of the treatment but who was smear- or culture-negative in the last month of treatment and on at least one previous occasion. Patients who completed treatment but who did not have a negative sputum smear or culture result in the last month of treatment and on at least one previous occasion were classified as having “completed treatment”. These two categories were considered as treatment success [17].

A homeless person was defined as a person living on the street or in municipal facilities (with no fixed abode) and who could be at risk of homelessness at any moment.

Prison or legal problems were defined as persons with a history of incarceration in jail.

A smoker was defined as any person smoking any number of cigarettes every day during the last month, including one.

Alcohol abuse was defined as alcohol consumption above 280 g per week in men and over 170 g in women.

Substance abuse was defined as harmful or hazardous use of psychoactive substances.

### Information sources and variables

Information on cases was obtained from the clinical histories at Serveis Clínics. Cases were followed-up by consulting the clinical histories at Serveis Clínics, the registry of TB cases of the TB Control Programs of Barcelona and Catalonia, the Shared Clinical History of Catalonia, the Central Registry of Insured Persons, and the Barcelona census. Epidemiological information on the disease and the sociodemographic characteristics of cases were completed by using these registries. The final follow-up date and status of TB patients who had left Catalonia or had died were also obtained from these registries.

The dependent variable was the presence or absence of recurrence. The independent variables were age (tertiles), sex, country of origin (Spain/outside Spain), homelessness, prison or legal problems, smoking, alcohol abuse, substance abuse, HIV infection, hepatitis C, diabetes, pulmonary comorbidities, type of TB (pulmonary, extrapulmonary), chest x-ray (presence or absence of cavitations), resistances, type of resistance (rifampicin, other drugs), treatment length, and prior TB episodes.

### Ethical considerations

All data were fully anonymized and were kept strictly confidential following the ethical principles of the Declaration of Helsinki [18], the Spanish Data Protection Law 3/2018 [19], and the General Data Protection Regulation (EU) No. 2016/679 [20]. The project was approved by the ethics committee for clinical research of Vall d’Hebron University Hospital in Barcelona (no. PR(AG)19/2016).

### Data analysis

We analyzed the distribution of frequencies and calculated measures of central tendency, the median and interquartile range (IQR). Univariate analysis was conducted with quantitative and qualitative variables.

Incidence density was calculated both overall and stratified by age group, country of origin, smoking, alcohol abuse, HIV infection, type of TB, prior episode of TB, and resistance to at least one drug. Incidence density was expressed in 100 person-years of follow-up. The numerator was the total number of TB recurrences in the study period and the denominator was the sum of all the individual follow-up periods.

We calculated the cumulative incidence of TB recurrences at 1, 2, 5, and 10 years of follow-up with 95% confidence intervals (95%CI). The risk of recurrence at 5 years was calculated according to the various independent variables. The cumulative incidence of TB recurrence was estimated by Kaplan-Meier curves both overall and stratified by country of origin, smoking, age group, and prior TB episodes. Groups were compared using the log rank test, taking a P-value less than 0.05 as statistically significant.

Based on Cox proportional hazards models, crude and adjusted hazard ratios (HR) for the risk of recurrence and their 95% CI were calculated by multivariate analysis. A model was created with all the variables and those with a P-value higher than 0.2 in the bivariate analysis were progressively excluded. For each variable included in the model, we confirmed that the risks were proportional through Shoenfeld’s partial residuals. To compare the Cox proportional hazards models, we used Akaike’s information criterion and Bayesian information criterion.

The statistical analysis was performed using the SPSS25 and STATA (Versión 13.0; Stata Corp, College Station, TX) programs.

## Results

Between 2000 and 2016, a total of 1064 patients with TB were admitted to Serveis Clínics. Of these, we excluded 225 patients from the cohort (55 lost to follow-up during treatment, 20 moved home, 77 had no health card number, and 73 did not have a minimum of 1 year of follow-up after cure). In all, 839 successfully treated patients who were disease-free at a minimum of 1 year were followed-up (S1 Fig).

Among the 839 patients, the median age was 40 years (IQR, 31–50), 727 (86.7%) were men and 467 (55.7%) were immigrants. A total of 158 (18.8%) were homeless, 74 (8.8%) had been in prison or had legal problems, 583 (69.5%) smoked, 379 (45.2%) showed risky alcohol consumption, and 171 (20.4%) were drug users. In all, 185 (22.1%) had HIV infection and 141 (16.8%) had hepatitis C infection. Most (88.0%) had pulmonary TB, 40.9% had cavities on chest x-ray, and 13.6% were resistant to at least one drug. There were 24 recurrences (2.9%) during the follow-up period (2000–2017) (Table 1).

**Table 1:** Probability of recurrence by various sociodemographic, epidemiological and clinical factors in the 2000–2016 tuberculosis cohort of Serveis Clínics, Catalonia.

Variables	Categories	Cases (N = 839) N (%)	Recurrences (N = 24) N (%)	No reccurrences (N = 815) N (%)	cHR (95%CI)	P-value	aHR (95%CI)**	P-value
**Age**	Median	40 (31–50)	43.5 (37–51)	40 (31–49)				
**Age groups**	15–34	296 (35.3)	3 (12.5)	293 (36.0)	1		1	
** **	35–45	263 (31.3)	11 (45.8)	252 (30.9)	4.41 (1.23–15.80)	0.023	3.90 (1.06–14.34)	0.04
** **	>45	280 (33.4)	10 (41.7)	270 (33.1)	3.81 (1.05–13.86)	0.042	3.88 (1.02–14.80)	0.047
**Sex**	Men	727 (86.7)	23 (95.8)	704 (86.4)	3.75 (0.51–27.80)	0.195		
** **	Women	112 (13.3)	1 (4.2)	111 (13.6)	1			
**Country of origin**	Spain	372 (44.3)	15 (62.5)	357 (43.8)	2.06 (0.90–4.71)	0.086		
	Outside Spain	467 (55.7)	9 (37.5)	458 (56.2)	1			
**Homelessness**	No	681 (81.2)	21 (87.5)	660 (81.0)	1			
** **	Yes	158 (18.8)	3 (12.5)	155 (19.0)	0.69 (0.21–2.33)	0.553		
**Prison or legal problems**	No	765 (91.2)	22 (91.7)	743 (91.2)	1			
** **	Yes	74 (8.8)	2 (8.3)	72 (8.8)	0.97 (0.23–4.14)	0.971		
**Smoking**	No	256 (30.5)	2 (8.3)	254 (31.2)	1		1	
** **	Yes	583 (69.5)	22 (91.7)	561 (68.8)	4.54 (1.07–19.32)	0.04	3.51 (0.80–15.37)	0.095
**Alcohol abuse**	No	460 (54.8)	9 (37.5)	451 (55.3)	1			
** **	Yes	379 (45.2)	15 (62.5)	364 (44.7)	1.93 (0.85–4.42)	0.118		
**Substance abuse**	No	668 (79.6)	18 (75.0)	650 (79.8)	1			
** **	Yes	171 (20.4)	6 (25.0)	165 (20.2)	1.29 (0.51–3.25)	0.588		
**HIV infection**	No	654 (77.9)	17 (70.8)	637 (78.2)	1			
** **	Yes	185 (22.1)	7 (29.2)	178 (21.8)	1.52 (0.63–3.68)	0.348		
**Hepatitis C**	No	698 (83.2)	18 (75.0)	680 (83.4)	1			
** **	Yes	141 (16.8)	6 (25.0)	135 (16.6)	1.76 (0.70–4.43)	0.231		
**Diabetes**	No	788 (93.9)	21 (87.5)	767 (94.1)	1			
** **	Yes	51 (6.1)	3 (12.5)	48 (5.9)	2.28 (0.68–7.64)	0.182		
**Pulmonary comorbidities**	No	740 (88.2)	21 (87.5)	719 (88.2)	1			
	Yes	99 (11.8)	3 (12.5)	96 (11.8)	1.16 (0.35–3.89)	0.809		
**Type of TB**	Extrapulmonary	101 (12.0)	5 (20.8)	96 (11.8)	1.90 (0.71–5.10)	0.201	2.57 (0.94–6.99)	0.065
	Pulmonary	738 (88.0)	19 (79.2)	719 (88.2)	1		1	
**Chest x-ray**	Absence of cavitations	496 (59.1)	16 (66.7)	480 (58.9)	1			
** **	Presence of cavitations	343 (40.9)	8 (33.3)	335 (41.1)	0.71 (0.30–1.65)	0.419		
**Resistance**	No	725 (86.4)	18 (75.0)	707 (86.7)	1		1	
** **	Yes	114 (13.6)	6 (25.0)	108 (13.3)	2.26 (0.90–5.71)	0.083	2.91 (1.11–7.65)	0.031
**Type of resistance**	No	725 (86.4)	18 (2.5)	707 (97.5)	1			
	Rifampicin	60 (7.2)	3 (5.0)	57 (95.0)	2.09 (0.61–7.08)	0.239		
	Other resistances	54 (6.4)	3 (5.6)	51 (94.4)	2.48 (0.73–8.41)	0.146		
**Treatment length**	6 months	397 (47.3)	7 (29.2)	390 (47.9)	1			
	9 months	238 (28.4)	11 (45.8)	227 (27.9)	2.76 (1.07–7.11)	0.036		
	12 months	70 (8.3)	1 (4.2)	69 (8.5)	0.83 (0.10–6.76)	0.863		
	18–24 months	134 (16.0)	5 (20.8)	129 (15.8)	2.28 (0.72–7.18)	0.16		
**Prior TB episodes**	No	673 (80.2)	15 (62.5)	658 (80.7)	1		1	
	Yes	166 (19.8)	9 (37.5)	157 (19.3)	2.49 (1.09–5.69)	0.031	2.12 (0.91–4.94)	0.081

** Schoenfeld Residuals Test chi-square = 6.13 p-value = 0.09

cHR: crude hazard ratio; aHR: adjusted hazard ratio; 95%CI: 95% confidence interval; HIV: human immunodeficiency virus; TB: tuberculosis.

The mean length of follow-up in the 839 patients was 5.81 years and the median was 4.74 years (IQR 2.20–9.01). Among the 839 TB cases in the cohort, the Incidence density of recurrence was 0.49 per 100 person-years (492.65 per 100,000 PY). There were no significant differences between the groups in the variables analyzed. Even so, comparison of Incidence density by age groups showed that it was higher among patients aged 35–45 years, followed by the group aged 45 years, and lastly by that aged 15–34 years. Incidence density was higher in patients born in Spain than in those born outside Spain, among smokers, patients with risky alcohol intake, and HIV-infected patients, as well as among patients with pulmonary TB, patients with resistance to at least one drug, and those with a prior episode of TB (Table 2).

**Table 2:** Incidence density of recurrence among tuberculosis patients in the 2000–2016 cohort of Serveis Clínics, Catalonia.

Variables	Categories	N	ID per 100 PY	95%CI
**Global**		839	0.49	0.33–0.74
**Age groups**	15–34	296	0.16	0.05–0.51
** **	35–45	263	0.73	0.40–1.32
** **	>45	280	0.66	0.35–1.22
**Country of origin**	Spain	372	0.66	0.40–1.10
** **	Outside Spain	467	0.35	0.18–0.66
**Smoking**	No	256	0.15	0.04–0.59
** **	Yes	583	0.63	0.41–0.95
**Alcohol abuse**	No	460	0.35	0.18–0.67
	Yes	379	0.65	0.39–1.08
**HIV infection**	No	654	0.45	0.28–0.72
** **	Yes	185	0.66	0.32–1.39
**TB localization**	Extrapulmonary	101	0.95	0.39–2.28
	Pulmonary	738	0.44	0.28–0.69
**Resistances**	No	725	0.42	0.27–0.67
** **	Yes	114	0.97	0.44–2.16
**Prior TB episodes**	No	673	0.39	0.23–0.64
** **	Yes	166	0.92	0.48–1.77

ID: incidence density; PY: person-years of follow-up; 95%CI: 95% confidence intervals; HIV: human immunodeficiency virus; TB: tuberculosis

The probability of recurrence in patients with TB admitted to Serveis Clínic and successfully treated was 0.63% (95% CI: 0.26–1.51) at the end of 1 year of follow-up, and was 1.35% at 2 years (95% CI:0.73–2.49), 3.69% at 5 years (95% CI: 2.45–5.54) and 3.98% at 10 years (95%CI: 2.65–5.95) (Table 3). No statistically significant differences were found in recurrence risk at 5 years among the variables analyzed (Table 4).

**Table 3:** Cumulative incidence of tuberculosis recurrences at 1, 2, 5, and 10 years of follow-up.

Time (years of follow-up)	Risk (%)	95%CI
1 year	0.63	0.26–1.51
2 years	1.35	0.73–2.49
5 years	3.69	2.45–5.54
10 years	3.98	2.65–5.95

95%CI: 95% confidence intervals.

**Table 4:** Percentage of recurrence risk according to various clinical-epidemiological factors at 5 years of follow-up among tuberculosis patients in the 2000–2016 cohort, Serveis Clínics, Catalonia.

Variables	Categories	Recurrences N = 24 (%)	Recurrence risk at 5 years (%)	95%CI
**Age groups (years)**	15–34	3 (12.5)	1.55	0.49–4.83
** **	35–45	11 (45.8)	5.61	3.11–9.99
** **	>45	10 (41.7)	4.21	2.18–8.03
**Sex**	Men	23 (95.8)	4.1	2.69–6.21
** **	Women	1 (4.2)	1.12	0.16–7.71
**Country of origin**	Spain	15 (62.5)	4.76	2.82–7.97
** **	Outside Spain	9 (37.5)	2.83	1.47–5.43
**Homelessness**	No	21 (87.5)	3.77	2.43–5.81
	Yes	3 (12.5)	3.49	1.11–10.7
**Prison or legal problems**	No	22 (91.7)	3.73	2.43–5.72
	Yes	2 (8.3)	3.08	0.77–11.82
**Smoking**	No	2 (8.3)	1.44	0.35–5.8
	Yes	22 (91.7)	4.62	3.02–7.04
**Alcohol abuse**	No	9 (37.5)	2.57	1.28–5.12
	Yes	15 (62.5)	5	3.02–8.24
**Substance abuse**	No	18 (75.0)	3.4	2.11–5.46
	Yes	6 (25.0)	4.85	2.16–10.71
**HIV infection**	No	17 (70.8)	3.22	1.97–5.25
	Yes	7 (29.2)	5.47	2.59–11.37
**Hepatitis C**	No	18 (75.0)	3.24	2.01–5.19
	Yes	6 (25.0)	6.2	2.74–13.74
**Diabetes**	No	21 (87.5)	3.4	2.19–5.26
	Yes	3 (12.5)	7.87	2.56–22.86
**Pulmonary comorbidities**	No	21 (87.5)	3.62	2.33–5.61
	Yes	3 (12.5)	4.09	1.32–12.28
**TB localization**	Extrapulmonary	5 (20.8)	7.77	3.23–18.07
	Pulmonary	19 (79.2)	3.13	1.97–4.96
**Chest x-ray**	Absence of cavitations	16 (66.7)	4.6	2.81–7.48
** **	Presence of cavitations	8 (33.3)	2.45	1.17–5.1
**Resistance**	No	18 (75.0)	3.17	1.96–5.11
** **	Yes	6 (25.0)	7.32	3.35–15.63
**Treatment length**	6 months	7 (29.2)	1.73	0.78–3.84
	9 months	11 (45.8)	7.04	3.91–12.53
	12 months	1 (4.2)	1.79	0.25–12.01
	18–24 months	5 (20.8)	4.86	2.03–11.39
**Prior TB episodes**	No	15 (62.5)	2.87	1.69–4.85
** **	Yes	9 (37.5)	7.01	3.67–13.18

95%CI: 95% confidence intervals; HIV: human immunodeficiency virus; TB: tuberculosis.

**Fig 1:**
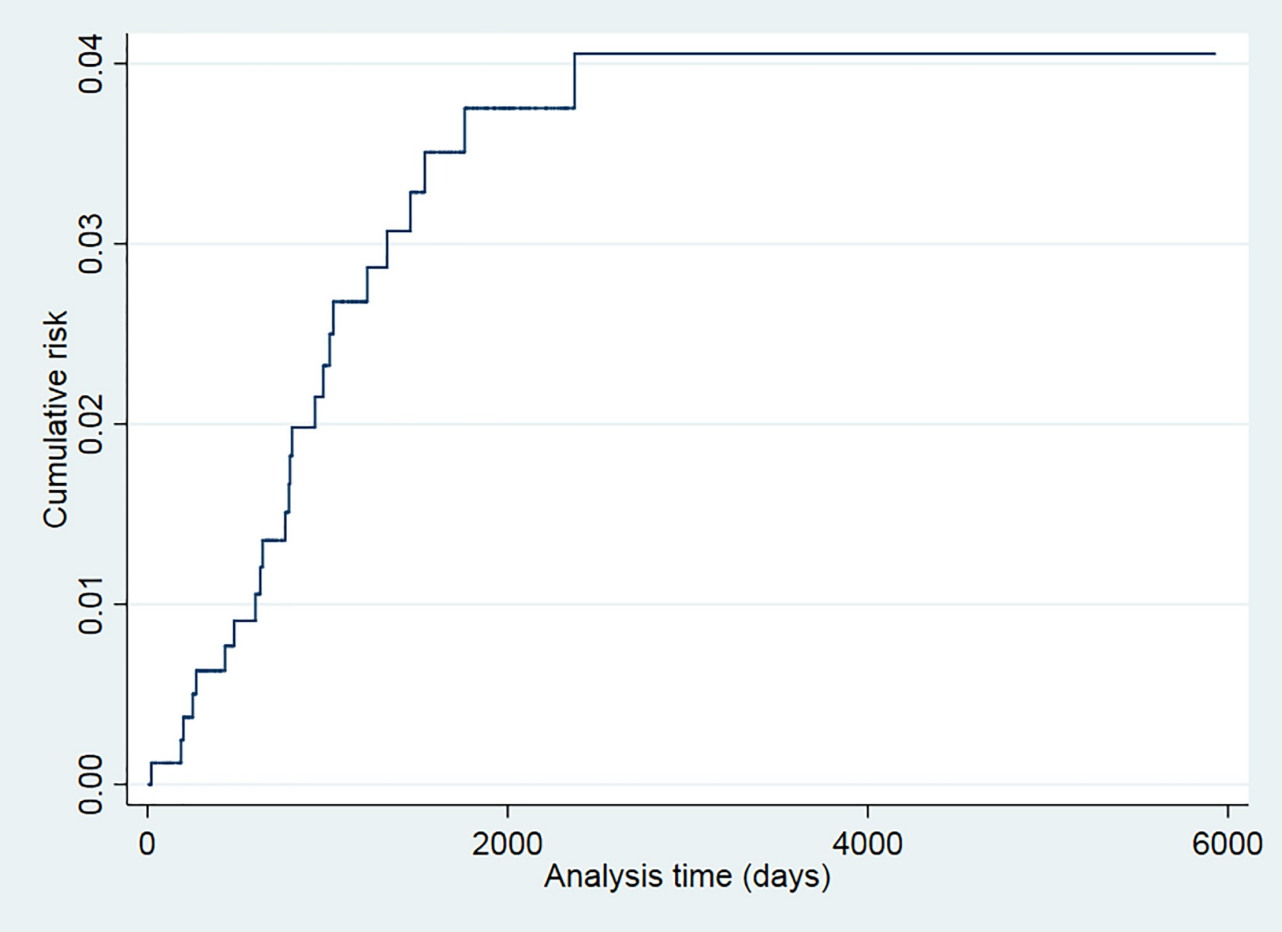
Kaplan-Meier curves of tuberculosis recurrence risk among all tuberculosis patients in the 2000–2016 cohort of Serveis Clínics, Catalonia.

**Fig 2:**
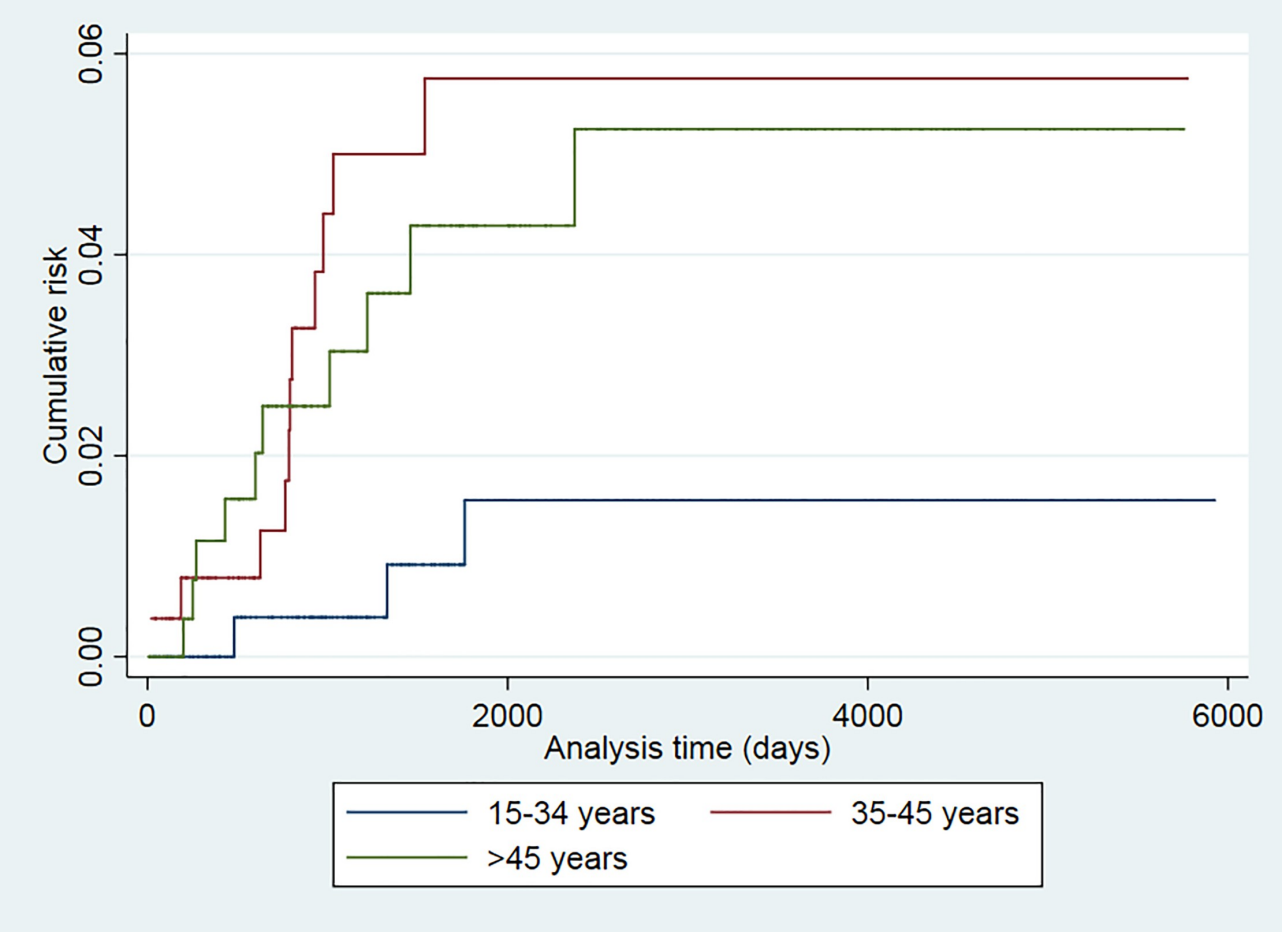
Kaplan-Meier curves of tuberculosis recurrence risk among all tuberculosis patients in the 2000–2016 cohort of Serveis Clínics, Catalonia, according to age group (p value Log rank test = 0.043).

**Fig 3:**
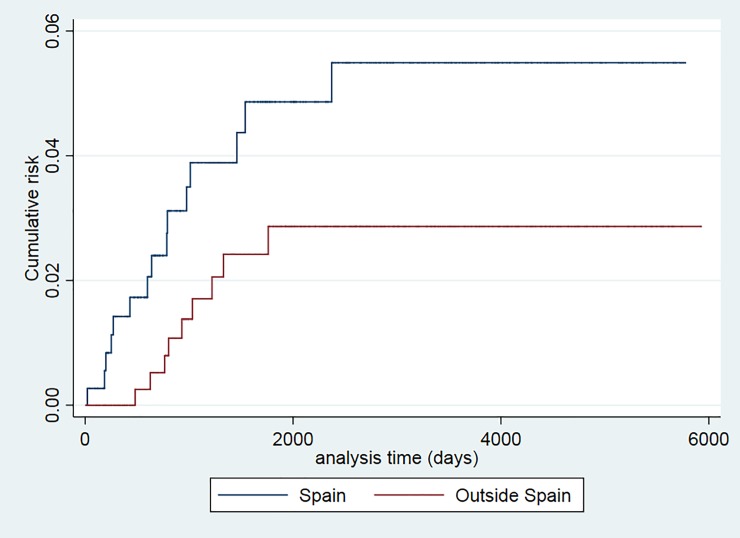
Kaplan-Meier curves of tuberculosis recurrence risk among all tuberculosis patients in the 2000–2016 cohort of Serveis Clínics, Catalonia, according to country of origin (p value Log rank test = 0.08).

**Fig 4:**
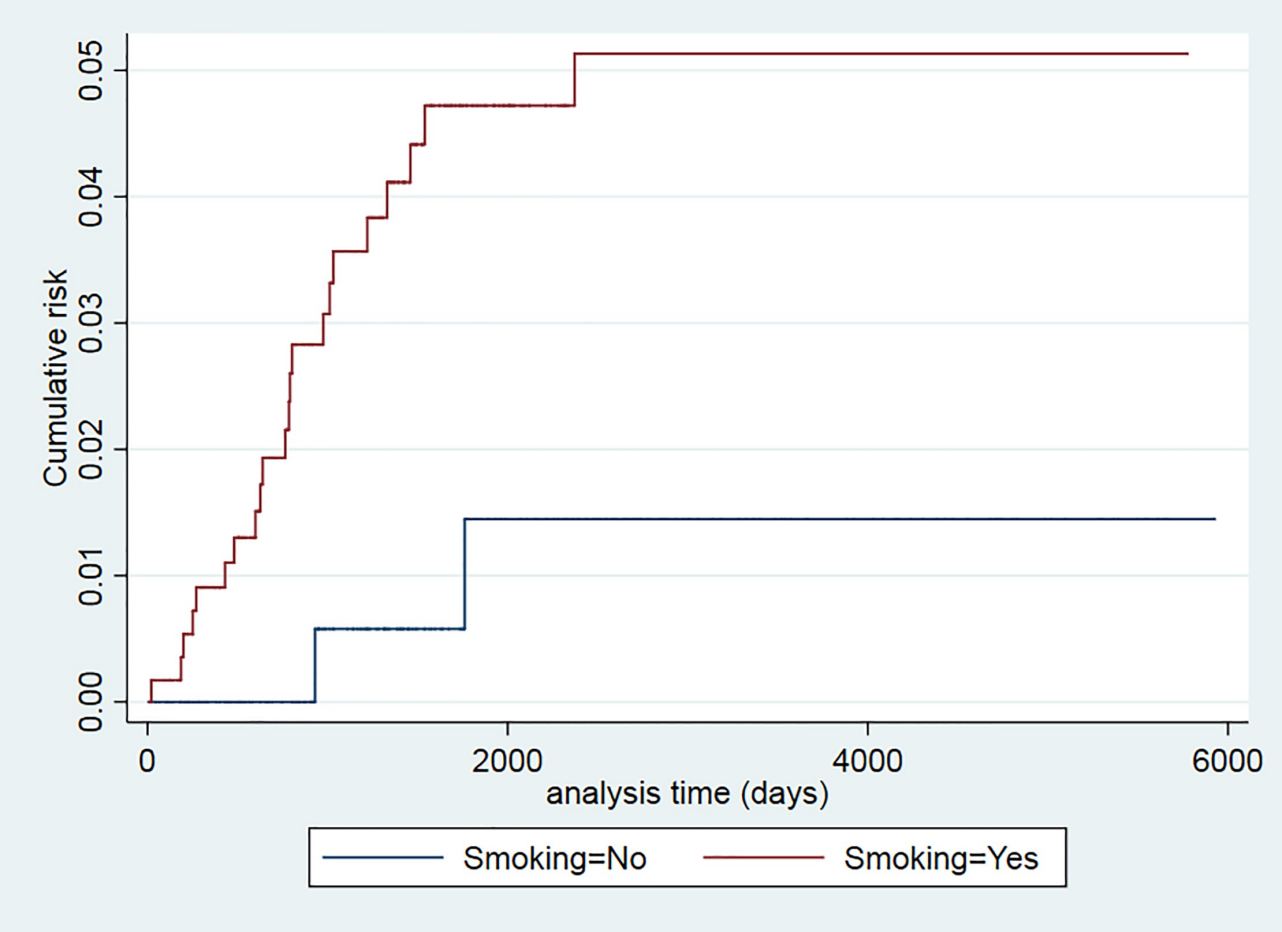
Kaplan-Meier curves of tuberculosis recurrence risk among all tuberculosis patients in the 2000–2016 cohort of Serveis Clínics, Catalonia, according to smoking (p value Log rank test = 0.024).

**Fig 5:**
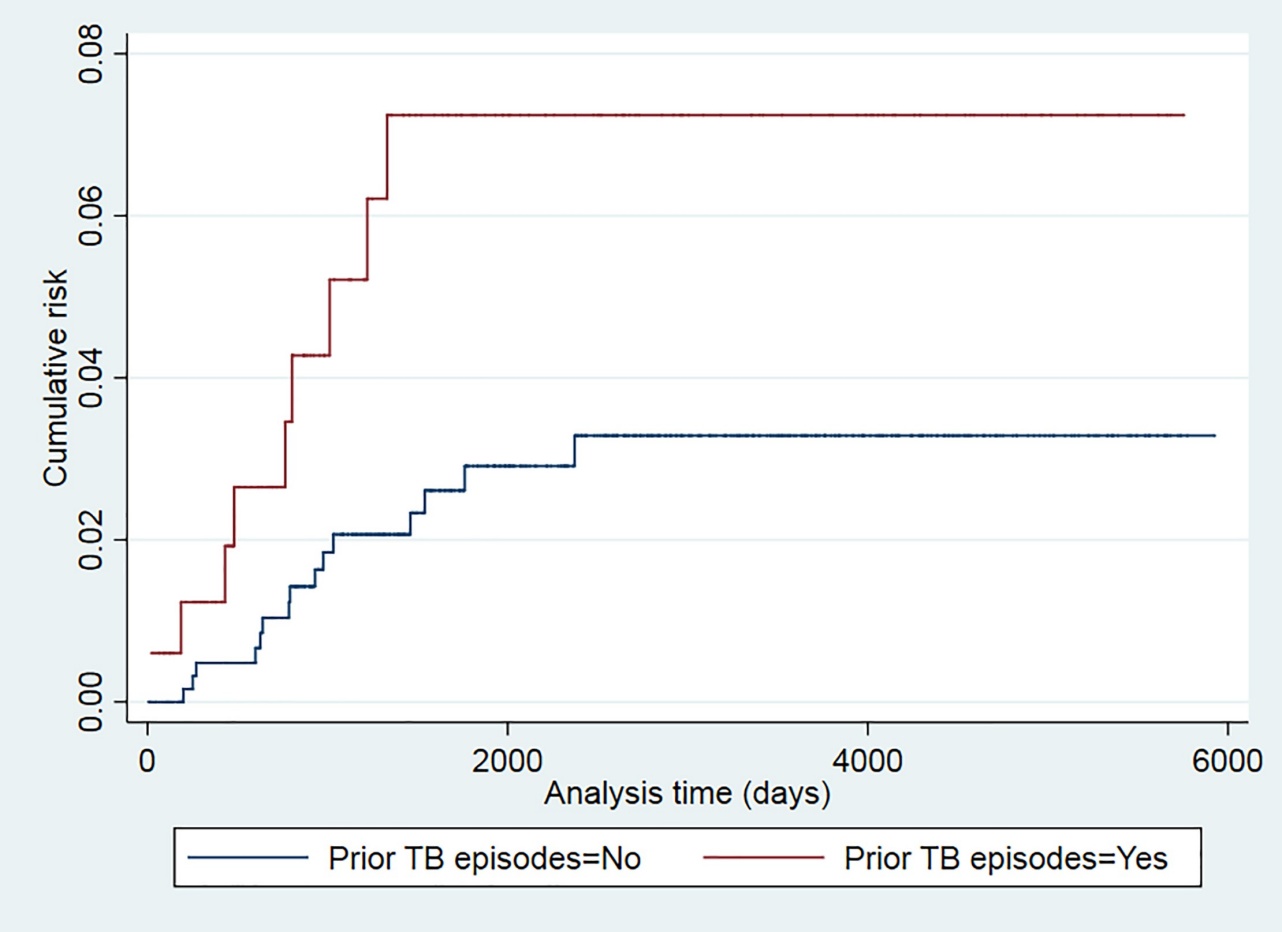
Kaplan-Meier curves of tuberculosis recurrence risk among all tuberculosis patients in the 2000–2016 cohort of Serveis Clínics, Catalonia, according to prior TB episode (p value Log rank test = 0.025).

**Fig 6:**
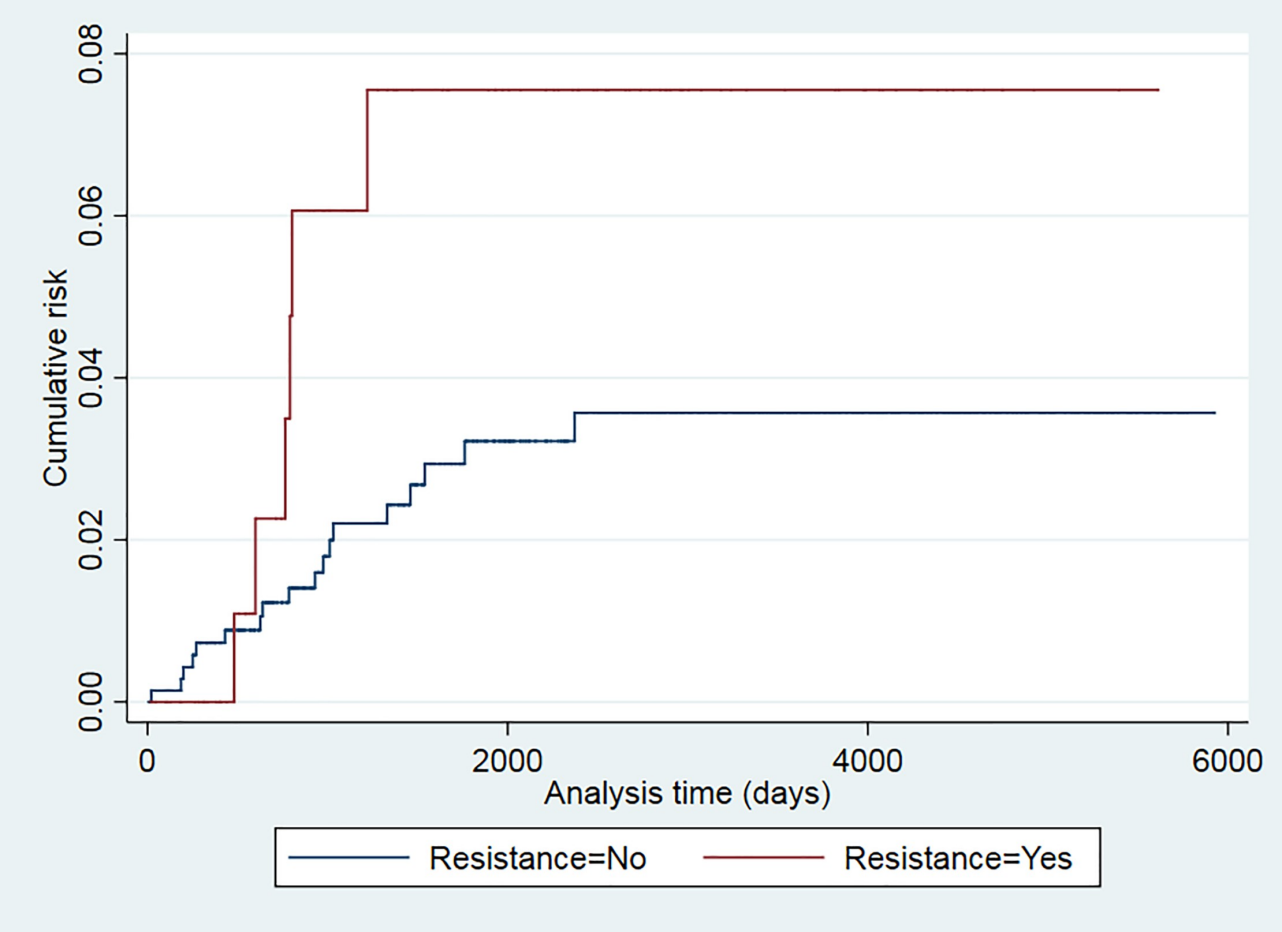
Kaplan-Meier curves of tuberculosis recurrence risk among all tuberculosis patients in the 2000–2016 cohort of Serveis Clínics, Catalonia, according to resistance (p value Log rank test = 0.075).

The recurrence risk was higher among smokers (p = 0.024), patients older than 34 years (p = 0.043), and those with a prior TB episode (p = 0.025) (Figs 1–6). The Schoenfeld test showed that the risks were proportional over time for all variables.

On univariate analysis, the factors predictive of a subsequentTB episode were age between 35 and 45 years (cHR: 4.406 CI: 1.229–15.795) and age older than 45 years (cHR: 3.814 CI: 1.049–13.859), smoking (cHR: 4.542 CI: 1.068–19.317), having received treatment for 9 months (cHR: 2.755 CI: 1.068–7.108), and having had a prior TB episode (cHR: 2.49 CI: 1.089–5.689) (Table 1).

On multivariate analysis, the factors predictive of recurrence were age older than 34 years (aHR = 3.90; CI = 1.06–14.34 in the group aged 35–45 years and aHR = 3.88; CI = 1.02–14.80 in that aged >45 years) and resistance to at least one anti-TB drug (aHR = 2.91; CI = 1.11–7.65) (Table 1).

## Discussion

Among the 839 TB cases in the cohort, there were 24 recurrences (2.9%), representing a rate of 0.49 per 100 person-years (95%CI: 0.33–0.74), and indicating that, for every 200 TB patients successfully treated and disease-free at 1 year, there was 1 recurrence. The factors predictive of recurrence among patients admitted to Serveis Clínics and successfully treated between 2000 and 2016 were age and drug resistance in the first TB episode.

Considering that this study was conducted in vulnerable individuals with risk factors, we believe that the incidence density of recurrences was low in comparison with incidence rates of TB recurrence reported by other studies conducted in the city of Barcelona (0.53 per 100 person-years in the period 1995–1997 and 0.34 per 100 person-years between 2003–2006 since one year after treatment completion) [14,21], in other European regions such as England and Wales (0.41 per 100 person-years after 12 months from the initial notification from 1998–2005) [22], New South Wales, Australia (71 per 100,000 person-years since treatment completion from 1994–2006) [23] and in countries with a low TB burden (1.8 per 100 person-years at 12 months of follow-up) [6].

This low recurrence rate, which is similar to recurrence rates reported in the general population, could indicate that recurrence is not related to the sociodemographic and clinical risk factors present in the population admitted to the TB clinic of Serveis Clínics. That is, that patients with risk factors for poor adherence have the same recurrence risk as the general population.

The incidence of TB in Catalonia during the study period oscillated between 27.6/100,000 inhabitants in the year 2000 and 13.3/100,000 inhabitants in 2016 [24]. The incidence rate for recurrence in the clinic was between 17 and 36 times higher than that for TB in the general population. As already indicated by other studies, the recurrence rate is higher than the rate of new TB cases [7]. A review by Rosser et al. also indicates that patients cured of a TB episode have a higher risk of TB than those without prior infection. These authors estimated that the TB rate was 31.5 times higher in recurrences than in new TB cases [5].

Recurrences include both endogenous reactivations and exogenous reinfections. Other studies performed in areas with a low burden of the disease have concluded that relapses of a prior infection continue to be the most common cause of TB recurrences. Infections with new *Mycobacterium tuberculosis* strains are possible but infrequent in low endemic areas [5,12,23,25,26]. Nevertheless, two studies conducted in Spain highlight the importance of TB infections in contexts with a low or moderate incidence [27,28]. El Sahly et al. also concluded that reinfection with a new *M*. *tuberculosis* strain caused a significant proportion of TB recurrences in an area with a low incidence [29]. The possibility of exogenous reinfection increases when the prevalence of the disease is higher [8,30,31].

In this study, factors predictive of recurrence were age between 35 and 45 years, age older than 45 years, and resistance to at least one anti-TB drug in the prior TB episode.

Other studies have reported a relationship between age and TB recurrences, although the results were not homogeneous. A study in England and Wales reported that children aged 0–14 years had a lower recurrence risk than patients aged 15–44 years [22]. In Brazil, the risk was lower in HIV-infected patients aged 40–49 years than in those aged <30 years [32]. In a 13-year cohort of TB cases in the United States, recurrence risk was higher in patients ≥ 65 years than in those aged <45 years [33]. A study performed in South Carolina also identified age > 46 years as a risk factor for recurrence [34]. In 1980 in the United States, TB recurrences were more common among patients aged > 30 years [35]. In South Africa, an association was found between advanced age at the first diagnosis of TB and recurrence in HIV-negative patients [36].

In addition to age, the other predictive factor for recurrence among TB patients treated in Serveis Clínics was resistance to at least one anti-TB drug. In China and Vietnam, an association was found between MDR and TB recurrence [37,38], while TB recurrences among South African miners were associated with anti-TB drug resistance [39]. In Uzbekistan, resistance to treatment was also found to substantially contribute to TB recurrences [40]. In southern India, isoniacide and/or rifamicin resistance was a predictive factor for recurrence among pulmonary TB patients under DOT [41].

In areas of low incidence, resistant TB was also identified as a risk factor for recurrence [5]. In California, pyrazinamide monoresistance was associated with TB recurrences [42]. Two studies performed in Italy showed a higher risk of recurrence versus reinfection in patients infected with MDR TB [25,43]. A meta-analysis concluded that one of the globally recognized risk factors for TB recurrence was multidrug resistance [44]. Moreover, among patient with resistant TB, recurrences were associated with a higher number of drugs and with resistance to all injectable drugs [45].

Among lifestyle factors, although smoking was not a predictive factor for recurrence in this study, smokers had a higher recurrence risk, with a lower CI limit close to 1, which was not significant, possibly due to a lack of statistical power.

In this regard, a study conducted in Taipei concluded that the risk of TB recurrence among persons smoking >10 cigarettes/day was twice that of nonsmokers and exsmokers [46]. Another study performed in the United Kingdom found that smoking was a predictive factor for TB recurrence [47]. In Yemen, patients smoking > 20 cigarrettes/day had a higher risk of TB recurrence than those smoking <20 cigarrettes/day [48]. In South India, smoking was a predictive factor for recurrence [41].

Few studies have reported an association between treatment length and recurrences. While a study conducted in Hong Kong showed that prolonging both the intensive phase of treatment and general treatment by 50% or more protected against recurrence [49], another sutdy conducted in South Carolina found an interraction between treatment length longer than 24 months and poor adherence, which was a predictive factor for recurrence [34]. In this line, Thomas et al. concluded that patients taking their medication irregularly had twice the probability of experiencing a relapse (aOR = 2.5; 95%CI 1.4–4.6) [41]. This could be a cause of the higher number of recurrences found in patients receiving treatment lasting for 9 months.

Few studies have analyzed the effect of a prior TB episode. A case-control study conducted in Vietnam concluded that one of the factors associated with recurrence was having reported a prior history of TB [38]. A previous study conducted in Barcelona reported that a history of TB treatment before study inclusion was a risk factor for recurrence [14].

Using Kaplan-Meier curves, this study also revealed a tendency by country of origin, with the percentage of recurrences being higher among Spanish-born patients. In line with these results, a study conducted in California found that US-born patients had a higher recurrence risk than those born outside the US [42]. In contrast, other studies reported an association between foreign-born patients or immigrants and TB recurrences [21,47,50]. It has been reported that immigrants usually have a higher association with TB recurrence caused by reinfection [25,43]. In the present study, some recurrences may not have been detected in patients born outside Spain if they left Catalonia or returned to their country of origin.

Finally, patients with a disease or receiving treatment altering immune system functioning are more susceptible to TB recurrence. However, in this study, no significant association was found between diseases or treatments that could compromise the immune system and TB recurrence.

This study could show a selection bias because we could not follow-up persons without a health card number and they were excluded from the study (S1 Table). These persons could have had different characteristics from those included in the study and more risk factors for recurrence. Comparison of sociodemographic, epidemiological and clinical factors between included and excluded persons revealed differences in the following variables: country of birth (the percentage of individuals born outside Spain was higher among excluded patients), homelessness, having been in prison or having problems with the law, treatment length, and prior TB treatment. Therefore, we may have underestimated some associations between these risk factors and TB recurrences.

We may also have missed recurrences that could have occurred outside Catalonia in those patients who moved during treatment, at the end of treatment, or during follow-up. For these patients we only have information on the final follow-up date and their status just before they left Catalonia. Nevertheless, a strength of the study is that we were able to detect all recurrences that occurred not only in Serveis Clínics but also throughout Catalonia.

Another limitation is the retrospective cohort design that did not consider variability over time among the independent variables. The study was based on the baseline variables of each patient gathered during the first TB episode. Finally, we could not ascertain whether recurrences were due to endogenous reactivation or exogenous reinfection.

The study cohort included all patients admitted to Serveis Clínics over a 16-year period and with a long follow-up. The data sources are both reliable and diverse, allowing us to compare and contrast the distinct sources and complete the database. To do this, we performed broad, in-depth field work. We assessed the data quality, including the search and identification of missing data to complete the database.

Another strength of the study is its setting, especially the good functioning of the TB Program of Barcelona and Catalonia and Serveis Clínics, which is specialized in TB, has more than 25 years’ experience, and closely follows patients up until cure. DOT is carried out daily in all patients.

## Conclusions

This study shows that patients successfully treated in a TB referral clinic specialized in vulnerable individuals have a not neglectablerecurrence risk, which is higher than that of TB disease in the general population. More specifically, patients with older age and with resistance seem to be at particularly high risk. These findings support the importance of close follow-up of persons with a prior TB episode and of devoting surveillance resources to these individuals because, despite successful treatment, they have a high risk of recurrent disease.

## Supporting information

S1 Fig Flowchart of tuberculosis patients in Serveis Clínics, Catalonia, 2000–2016. (DOCX)

S1 Table Sociodemographic and clinical characteristics of excluded and included tuberculosis patients in the 2000–2016 cohort, Serveis Clínics, Catalonia. (DOCX)
