# Supplementary material for: Tuberculosis recurrences and predictive factors in a vulnerable population in Catalonia
Source: PLoS One. 2020 Jan 15;15(1):e0227291. doi: 10.1371/journal.pone.0227291 (PMC6961944; doi:10.1371/journal.pone.0227291)
Supplement: S1 Fig — (DOCX) [file pone.0227291.s001.docx]

**S1 Fig. Flowchart of tuberculosis patients in Serveis Clínics, Catalonia, 2000-2016**

**TB CASES ADMITTED IN SERVEIS CLÍNICS CATALUNYA 2000-2016: 1064**

**TB CASES SUCCESSFULLY TREATED: 989**

**TB CASES SUCCESSFULLY TREATED AND DISEASE-FREE FOR A MINIMUM OF 1 YEAR AFTER THE END OF TREATMENT: 839**

**24 TB RECURRENCES**

**Excluded: 75**

Lost to follow-up: 55

Transfers: 20

**Excluded: 150**

Cases without health card number: 77

Cases with follow-up less than 1 year from the end of treatment: 73
