# Supplementary material for: Tuberculosis recurrences and predictive factors in a vulnerable population in Catalonia
Source: PLoS One. 2020 Jan 15;15(1):e0227291. doi: 10.1371/journal.pone.0227291 (PMC6961944; doi:10.1371/journal.pone.0227291)
Supplement: S1 Table — (DOCX) [file pone.0227291.s002.docx]

| **S1 Table. Sociodemographic and clinical characteristics of excluded and included tuberculosis patients in the 2000-2016 cohort. Serveis Clínics. Catalonia** | | | | |
| --- | --- | --- | --- | --- |
| **Variables** | **Categories** | **Excluded (N=225)**  **N (%)** | **Included (N=839)**  **N (%)** | **P-value** |
| **Age** | Median | 37 (31-47) | 40 (31-50) | 0.061^ |
| **Age groups** | 15-34 | 84 (37.3) | 296 (35.3) |  |
|  | 35-45 | 80 (35.6) | 263 (31.3) |  |
|  | >45 | 61 (27.1) | 280 (33.4) | 0.187 |
| **Sex** | Men | 202 (89.8) | 727 (86.7) |  |
|  | Women | 23 (10.2) | 112 (13.3) | 0.211 |
| **Country of origin** | Spain | 62 (27.6) | 372 (44.3) |  |
|  | Outside Spain | 163 (72.4) | 467 (55.7) | <0.001 |
| **Homelessness** | No | 169 (75.1) | 681 (81.2) |  |
|  | Yes | 56 (24.9) | 158 (18.8) | 0.044 |
| **Prison or legal problems** | No | 187 (83.1) | 765 (91.2) |  |
|  | Yes | 38 (16.9) | 74 (8.8) | <0.001 |
| **Smoking** | No | 63 (28.0) | 256 (30.5) |  |
|  | Yes | 162 (72.0) | 583 (69.5) | 0.465 |
| **Alcohol abuse** | No | 117 (52.0) | 460 (54.8) |  |
|  | Yes | 108 (48.0) | 379 (45.2) | 0.45 |
| **Substance abuse** | No | 166 (73.8) | 668 (79.6) |  |
|  | Yes | 59 (26.2) | 171 (20.4) | 0.059 |
| **HIV infection** | No | 173 (76.9) | 654 (77.9) |  |
|  | Yes | 52 (23.1) | 185 (22.1) | 0.734 |
| **Hepatitis C** | No | 189 (84.0) | 698 (83.2) |  |
|  | Yes | 36 (16.0) | 141 (16.8) | 0.773 |
| **Diabetes** | No | 208 (92.4) | 788 (93.9) |  |
|  | Yes | 17 (7.6) | 51 (6.1) | 0.421 |
| **Pulmonary comorbidities** | No | 199 (88.4) | 740 (88.2) |  |
|  | Yes | 26 (11.6) | 99 (11.8) | 0.92 |
| **Type of TB** | Extrapulmonary | 33 (14.7) | 101 (12.0) |  |
|  | Pulmonary | 192 (85.3) | 738 (88.0) | 0.291 |
| **Chest x-ray** | Absence of cavitations | 129 (57.3) | 496 (59.1) |  |
|  | Presence of cavitations | 96 (42.7) | 343 (40.9) | 0.629 |
| **Resistance** | No | 189 (84.4) | 725 (86.4) |  |
|  | Yes | 35 (15.6) | 114 (13.6) | 0.435 |
| **Treatment length** | 6 months | 134 (69.4) | 397 (47.3) |  |
|  | 9 months | 42 (21.8) | 238 (28.4) |  |
|  | 12 months | 2 (1.0) | 70 (8.3) |  |
|  | 18-24 months | 15 (7.8) | 134 (16.0) | <0.001 |
| **Prior TB episodes** | No | 166 (73.8) | 673 (80.2) |  |
|  | Yes | 59 (26.2) | 166 (19.8) | 0.036 |

^Median test

HIV: Human immunodeficiency virus; TB: Tuberculosis.
